# The Relationships Between Nonsuicidal Self-Injury, Connectedness, and Suicide Risk in Youth Presenting to the Emergency Department

**DOI:** 10.1016/j.jaacop.2025.01.001

**Published:** 2025-01-14

**Authors:** Tesia Shi, Ritika Merai, Nathan J. Lowry, Donna A. Ruch, Jeffrey A. Bridge, Maryland Pao, Lisa M. Horowitz

**Affiliations:** a Intramural Research Program, National Institute of Mental Health, National Institutes of Health, Bethesda, Maryland; b Teachers College, Columbia University, New York, New York; c Ohio State University College of Medicine, Columbus, Ohio; d Abigail Wexner Research Institute, Nationwide Children’s Hospital, Columbus, Ohio

**Keywords:** adolescent, connectedness, emergency department, nonsuicidal self-injury, suicide risk

## Abstract

**Objective:**

Nonsuicidal self-injury (NSSI), defined as harming one’s own body without suicidal intent, is a strong risk factor for suicidal thoughts and behaviors in adolescents. One promising protective factor that can mitigate suicide risk conferred by NSSI is connectedness. This study aimed to examine the association between connectedness (family, school, peer, and overall), NSSI, and suicide risk in pediatric emergency department patients.

**Method:**

This is a secondary analysis of data from the Emergency Department Screen for Teens at Risk for Suicide (ED-STARS) study. Youth ages 12 to 17 years completed questions on demographics; past-year NSSI history; the Ask Suicide-Screening Questions (ASQ) tool; and 2 items each on family, school, and peer connectedness. Overall connectedness was a composite of the 6 items. Multivariable logistic regressions assessed the associations between connectedness, NSSI, and suicide risk.

**Results:**

Data were analyzed from 5,406 participants (55.2% female, 45.9% non-Hispanic White, mean [SD] age = 15.0 [1.7]). Out of all participants, 23.3% (1,258/5,406) screened positive for suicide risk, and 18.1% (981/5,406) reported past-year NSSI history. For every 1-point increase from mean family connectedness, the odds of screening positive for suicide risk decreased by 62% (odds ratio 0.38, 95% CI 0.31-0.46) for youth with NSSI and 70% (odds ratio 0.30, 95% CI 0.27-0.34) for youth without NSSI (difference: *z* = 1.96, *p* = .05).

**Conclusion:**

Connectedness was protective against suicide risk across the sample regardless of NSSI history. However, family connectedness was less protective for youth with NSSI compared with youth without NSSI. Future research should examine connectedness in greater detail and the quality of different relationships that could be protective for at-risk youth.

## Plain language summary

Nonsuicidal self-injury (NSSI), the act of harming one’s own body without suicidal intent, is a strong risk factor for suicidal thoughts and behaviors (STBs) in adolescents. One important protective factor that can decrease suicide risk and NSSI is connectedness. This study aimed to examine relations among types of connectedness (family, school, peer and overall), NSSI, and suicide risk in pediatric emergency department patients. All domains of connectedness (family, school, peer and overall) were found to be associated with lower suicide risk, regardless of NSSI history. However, family connectedness was associated with less of a buffering effect for youth with NSSI compared to youth without NSSI. System-level interventions that strengthen family, school, and peer networks have the potential to reduce youth suicidality risk.

Suicide is the second leading cause of death for youth ages 12 to 17 years, with rates rising in the last decade.^1^ In recent years, 22.2% of high school students and 22.3% of middle school students seriously considered attempting suicide.^2^^,^^3^ Adolescents at risk of suicide often engage in other risk behaviors such as substance abuse, interpersonal violence, and self-injury.^4^^,^^5^

Nonsuicidal self-injury (NSSI), defined as harming one’s own body without suicidal intent, is one of the strongest risk factors for suicidal thoughts and behaviors (STBs), even when controlling for other risk factors such as mental health disorders.^6^ Common NSSI methods include cutting and burning. Different characteristics of NSSI such as increased frequency,^7^ a wider set of methods,^8^ and earlier age of onset^8^ are also significantly associated with increased STBs. Prevalence rates of NSSI are highest among adolescents at 20%,^9^ increasing from age 12 onward and peaking at ages 15 to 17.^10^

Over the last decade, adolescent visits to the emergency department (ED) for STBs and/or NSSI have increased significantly by 2.5- to 5-fold.^11^^,^^12^ Suicide risk screening has shown that many adolescents who present for a medical chief complaint in an ED are also at risk of suicide.^13^ These rates, coupled with the finding that more than 1 in 3 youths who died by suicide had visited the ED in the months before their death, positions the ED as a critical avenue for mental health triaging and suicide prevention.^14^ Despite the strong association between NSSI and suicide risk, there is limited research on protective factors that can mitigate the suicide risk conferred by NSSI. Identifying which factors to intervene in to decrease STBs in these high-risk youth populations can better inform interventions in medical settings.

The interpersonal theory of suicide posits that an individual must have both the desire (thwarted belongingness and perceived burdensomeness) and the capacity for suicide before making a lethal suicide attempt.^15^ Although NSSI is theorized to primarily contribute to increased capacity due to increased pain tolerance, other research has shown that NSSI is associated with increased suicidal desire, specifically thwarted belongingness.^16^ Thus, one important protective factor and intervention target that can address thwarted belongingness and thus decrease an individual’s suicide risk may be connectedness.

Connectedness, which can be defined as feelings of belonging as well as the presence of close relationships, has been shown to be a strong protective factor against suicide^17^ and NSSI in youth.^18^ Prior studies about the associations between connectedness and risk behaviors in youth have focused primarily on family, school, and peer relationships. During adolescent development, these interpersonal relationships play a vital role in shaping identity development and reducing negative outcomes such as crime and homelessness.^19^ Feeling connected to others can also increase key protective processes such as self-regulation and coping; thus, system-level interventions that strengthen family, school, and peer networks may help reduce suicidal thoughts and behavior.^20^ Although past research in ED youth populations have examined NSSI and connectedness separately in relation to suicide risk,^21^ there has been limited research on the interaction between the 2 variables.

Family connectedness can be defined as feeling cared for and respected by one’s parents.^22^ Adolescents who have higher levels of family connectedness have lower levels of suicidal ideation,^22^ decreased odds of suicide attempt history,^23^ decreased NSSI severity,^22^ and increased coping.^24^ Connections to parents and nonparental adults may also be moderators between the association between risk factors and STBs in high-risk youth.^25^

School connectedness is defined as feelings of closeness and belonging in the school setting and includes relationships with teachers, school staff, and peers.^26^ It is protective against a range of risk behaviors including suicide attempts^27^ and suicide ideation.^23^ Increased family and school connectedness may also exert protective effects by addressing underlying issues that contribute to NSSI and suicide risk such as poor familial relationships and issues in the school setting such as bullying.^28^^,^^29^

Finally, peer connectedness is defined as feelings of support and trust in friends and peers.^22^ In contrast to school and family connectedness, connectedness to peers can have both positive and negative effects on STBs and NSSI depending on the nature of the relationship. Higher levels of peer connectedness not only are associated with decreased odds of attempting suicide and suicidal ideation,^30^ but also have been associated with an increased likelihood of suicidal ideation potentially due to peers also showing maladaptive behaviors.^31^ Although connections to friends can have protective effects, youth who engage in self-harm behaviors tend to have peers who also practice self-harm, possibly resulting in a contagion effect of NSSI and an increase in NSSI behavior.^25^^,^^32^

The primary aim of this exploratory study was to describe the associations between domains of connectedness (overall, family, school, and peer), NSSI, and suicide risk, particularly how the interaction between connectedness and NSSI contributes to suicide risk. We hypothesized that the interaction between overall, family, and school connectedness and NSSI would be significant. Due to research showing that the effects of peers can be complex depending on the nature of the relationship, we hypothesized that peer connectedness would not have a significant interaction with NSSI.

## Method

### Sample

This is a secondary analysis of study 1 of the Emergency Department Screen for Teens at Risk for Suicide (ED-STARS) study, which is an instrument development study for the Computerized Adaptive Screen for Suicidal Youth (CASSY) involving multiple EDs.^33^ The original study aimed to examine whether a computerized adaptive screen could predict youth suicide attempts at 3 months after ED discharge. Between June 2015 and July 2016, participants ages 12 to 17 years were recruited from 13 EDs in the United States that were part of the Pediatric Emergency Care Applied Research Network (PECARN). Participants who were medically unstable or severely cognitive impaired, non-English speaking, or a ward of the state were ineligible for the study. Following enrollment, participants completed a self-report survey on computer tablets that consisted of 92 primary questions and up to 27 additional questions.

Participants were included in the present exploratory analysis if they completed both items on each of the school, family, and peer connectedness scales, demographic questions, and the past-year NSSI item. If participants had an incomplete response on the Ask Suicide-Screening Questions (ASQ) tool but indicated a positive response to at least 1 of the 4 items, they were included in the sample as a positive screen. Any other incomplete ASQ responses were excluded (eg, answering “no” to a question but not completing all the questions). All data were collected from youth participant responses except race and ethnicity, which included parent responses about their child’s race/ethnicity.

### Measures

#### Demographic Information

Age and gender were measured by participant responses, whereas race and ethnicity were measured by both participant and parent/guardian self-reports due to significant missing racial data in the participant responses. Due to low cell sizes, for gender identity, if participants selected any option other than “male” and “female,” they were considered a gender minority (includes transgender male, transgender female, genderqueer, other). For the same reason, participants who reported being Asian or Native Hawaiian/Other Pacific Islander were grouped into Asian American Native Hawaiian Pacific Islander. If there was more than 1 race reported (eg, Black and White, White and Asian American Native Hawaiian Pacific Islander), participants were categorized as non-Hispanic multiracial. Participants who reported being Hispanic/Latino were categorized as Hispanic/Latino.

#### Nonsuicidal Self-Injury

Past-year NSSI was measured with 1 item (“In the past 12 months, have you ever harmed or hurt your body on purpose, such as cutting or burning your skin, or hitting yourself, without wanting to die”) adapted from the Youth Risk Behavior Survey.^34^ Descriptive data on past-week frequency (0 times, 1-2 times, 3-4 times, 5 or more times) and 11 methods of NSSI (items were adapted from the Functional Assessment for Self-Mutilation Scale^35^) including “other” were also reported.

#### Suicide Risk

Suicide risk was determined using the Ask Suicide-Screening Questions (ASQ) tool. The ASQ is a brief, 4-5 item screening instrument originally validated for use among pediatric ED patients.^36^ It has strong psychometric properties, with a sensitivity of 96.9% and specificity of 87.6% in the original study. A “yes” response to at least 1 of the items on the ASQ is considered a positive screen.

#### Connectedness

School connectedness was assessed using two 5-point Likert scale items (“You feel close to people at your school” and “You feel like you are part of your school”) adapted from the School Connectedness Scale.^26^ Responses ranged from “1—strongly disagree” to “5—strongly agree.” Family connectedness was assessed using two 5-point Likert scale items (“How much do people in your family understand you?” and “How much does your family pay attention to you?”) adapted from the Parent–Family Connectedness Scale.^26^ Responses ranged from “1—not at all” to “5—very much.” Peer connectedness was assessed using two 5-point Likert scale items (“I have friends I’m really close to and trust completely” and “Spending time with my friends is a big part of my life”) adapted from the Hemingway Measure of Adolescent Connectedness scale.^37^ Responses ranged from “1—not at all true” to “5—very true.”

The 2 items for each connectedness variable (school, family, and peer) were averaged into 1 composite value (range from low to high 1-5). Overall connectedness was derived from calculating a mean of the 6 items (range from low to high 1-5).^38^

#### Analytic Plan

Four multivariable logistic regression models (school, family, peer, overall) assessed the associations between each domain of connectedness, past-year NSSI, and binary suicide risk, with gender, race/ethnicity, and age as covariates. The primary outcome was suicide risk (positive/negative), with NSSI (yes/no), connectedness, and interaction between NSSI and connectedness as main effects. A significant interaction between NSSI and connectedness would indicate that the association between connectedness and suicide risk changes depending on NSSI presence. Continuous variables (connectedness and age) were mean centered. Descriptive statistics were calculated for sample characteristics. Significance was defined as *p* < .05, and all tests were 2-tailed. All analyses were conducted using R software version 4.3.1 (R Foundation for Statistical Computing, Vienna, Austria; https://www.r-project.org/).

## Results

### Sample Characteristics

Data were analyzed from 5,406 of 6,536 youth (83%) from the original sample. Most participants were female (55.2%) and non-Hispanic White (45.9%) with a mean (SD) age of 15 (1.7) years. Of the total sample, 23.3% (1,258 of 5,406) screened positive for suicide risk and 18.1% (981 of 5,406) reported NSSI history in the past year (Table 1). For youth who reported NSSI, 76.1% (747 of 981) also screened positive for suicide risk. For participants without past-year NSSI, 11.5% (511 of 4,425) screened positive for suicide risk.

**Table 1:** Participant Demographics

Demographics	Full sample (N = 5,406)			Past-year NSSI (n = 981)			Positive suicide risk screen (n = 1,258)			Overall connectedness	
	n	(%)		n	(%)		n	(%)		Mean	(SD)
Sample	—			981	(18.1)		1,258	(23.3)		3.94	(0.74)
Gender											
Male	2,301	(42.6)		250	(25.5)		310	(24.6)		4.07	(0.66)
Female	2,897	(55.2)		654	(66.7)		863	(68.6)		3.86	(0.77)
Gender minority	118	(2.2)		77	(7.8)		85	(6.8)		3.22	(0.87)
Race/ethnicity											
Hispanic or Latino	1,352	(25.0)		227	(23.1)		283	(22.5)		3.92	(0.73)
NH AANHPI	82	(1.5)		8	(0.82)		16	(0.50)		4.05	(0.59)
NH AIAN	40	(0.70)		5	(0.50)		7	(1.3)		3.88	(0.78)
NH Black	1,215	(22.5)		173	(17.6)		265	(21.1)		3.86	(0.74)
NH Multiracial	237	(4.4)		60	(6.22)		68	(5.4)		3.84	(0.77)
NH White	2,480	(45.9)		508	(51.8)		619	(49.2)		4.00	(0.75)
	**Mean**	**(SD)**	**[range]**	**Mean**	**(SD)**	**[range]**	**Mean**	**(SD)**	**[range]**		
Age, y	15.0	(1.7)	[12-17]	15.1	(1.5)	[12-17]	15.2	(1.5)	[12-17]	—	
	**n**	**(%)**		**n**	**(%)**		**n**	**(%)**			
Chief complaint											
Medical	4,655	(86.1)		536	(54.6)		661	(52.5)		4.01	(0.69)
Psychiatric	750	(13.9)		445	(45.4)		597	(47.5)		3.79	(0.82)
Unknown	1	(0.00)		0	(0.00)		0	(0.00)		5	

Note: Values for peer, school, and family connectedness are reported in Table S1, available online. AANHPI = Asian American Native Hawaiian Pacific Islander; AIAN = American Indian/Alaska Native; NH = non-Hispanic; NSSI = nonsuicidal self-injury.

**Figure 1:**
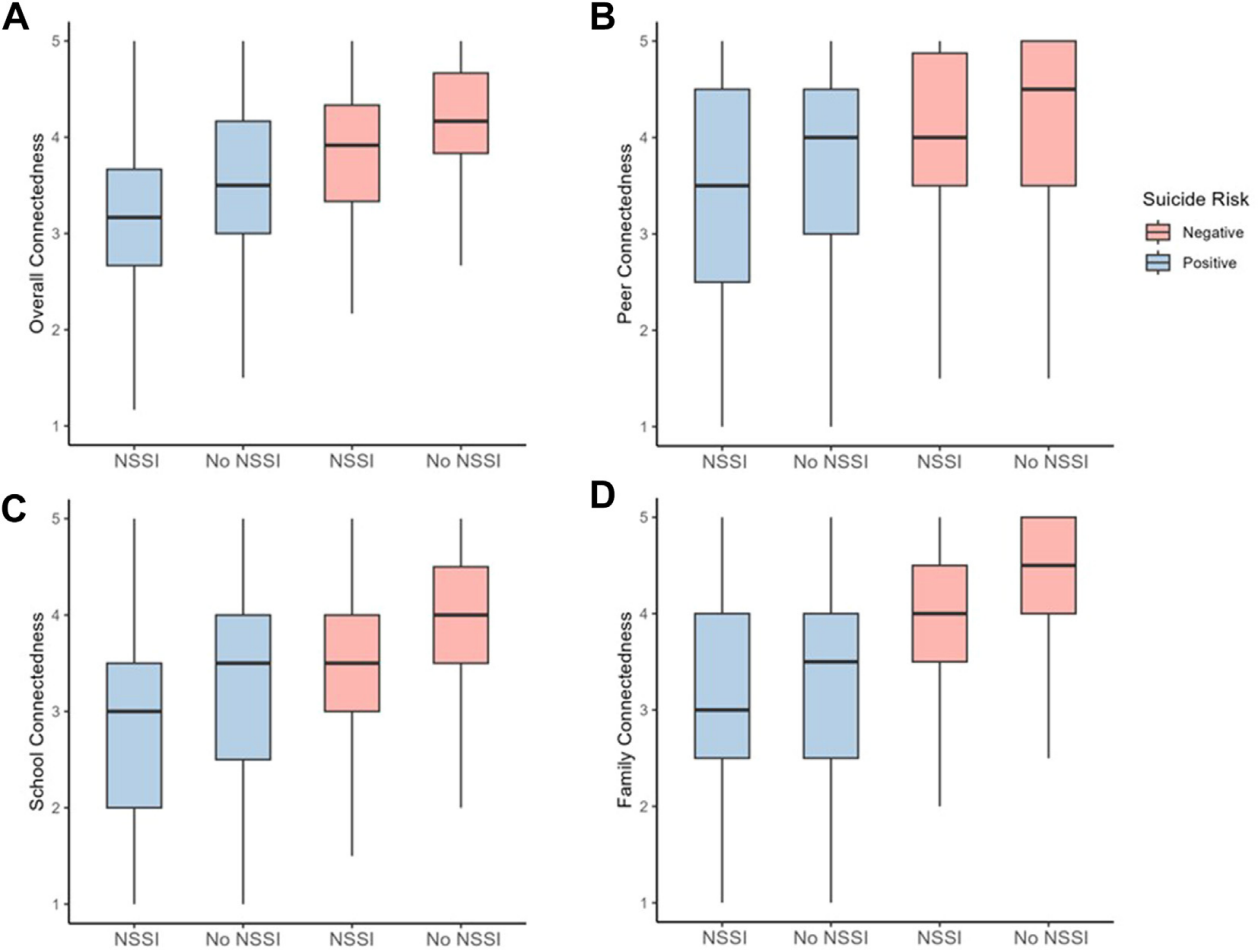
Connectedness by Nonsuicidal Self-Injury (NSSI) and Suicide Risk Groups

Gender minority youth had the highest rates of past-year NSSI (65.3%; 77 of 118) and screening positive for suicide risk (71.9%; 85 of 118) as well as the lowest mean overall connectedness score of 3.22 (range from low to high 1-5) compared with cisgender male and cisgender female adolescents. Multiracial youth had the highest rates of NSSI (25.3%; 60 of 237) and screening positive suicide risk (28.7%; 68 of 237) as well as the lowest mean overall connectedness score of 3.84 of 5 compared with other racial groups. Mean connectedness scores for peer, family, and school across demographic variables are reported in Table S1, available online. Youth who had both NSSI history and screened positive for suicide risk had the lowest median connectedness scores, followed by youth who screened positive for suicide risk but did not report NSSI (Figure 1).

### NSSI Sample Characteristics

Of youth who reported past-year NSSI, 37.6% (369 of 981) reported engaging in NSSI in the past week (Table 2). The most common methods were cutting or carving on skin (69.1%; 678 of 981), hitting self (39.4%; 387 of 981), and picking at a wound (39.4%; 387 of 981) of participants. For number of methods of NSSI, 56.6% (555 of 981) used multiple methods.

**Table 2:** Nonsuicidal Self-Injury (NSSI) Characteristics

Characteristic	Sample (N = 981)	
	n	(%)
Frequency (past year)		
1-2 times	451	(46.0)
3-4 times	187	(19.0)
≥5 times	343	(35.0)
Frequency (past week)		
0 times	606	(61.8)
1-2 times	246	(25.1)
3-4 times	69	(7.0)
≥5 times	54	(5.5)
Unknown	6	(0.60)
Method		
Cutting or carving on skin	678	(69.1)
Hitting self	387	(39.4)
Picking at a wound	387	(39.4)
Scraping skin	235	(24.0)
Biting self	181	(18.5)
Pulling out one’s own hair	124	(12.6)
Picking areas of the body to the point of drawing blood	117	(11.9)
Erasing skin to draw blood	76	(7.7)
Inserting objects under skin or nails	50	(5.1)
Tattooing, burning self	83	(8.5)
Other	105	(10.7)
No. methods		
1	410	(41.8)
>1	555	(56.6)
Unknown	16	(1.6)

Note: Participants can select multiple methods of NSSI, so percentages do not sum to 100.

### Logistic Regression Analysis

**Figure 2:**
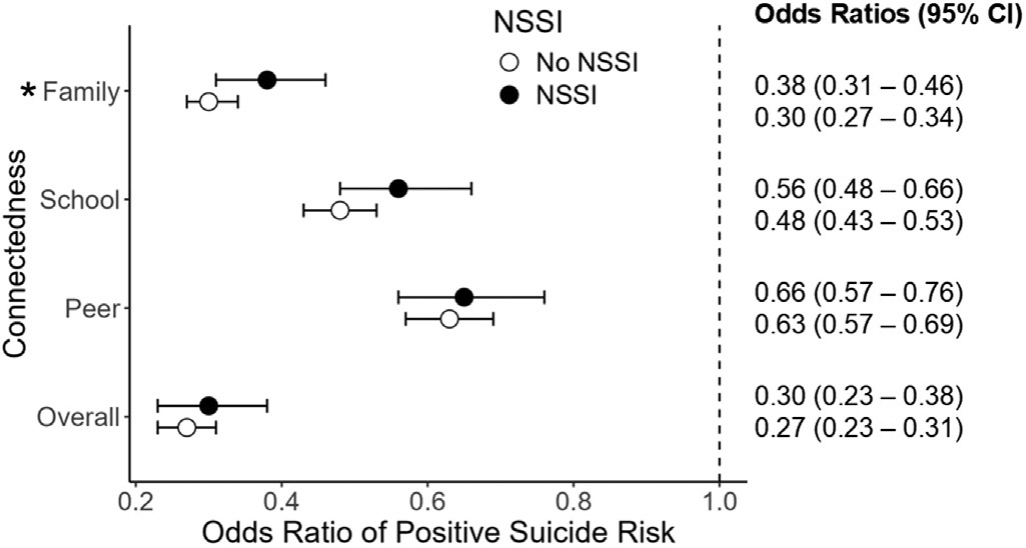
Odds Ratios for Effect of Connectedness on Suicide Risk in Nonsuicidal Self-Injury (NSSI) and No NSSI Groups ***Note:****In the interaction term between NSSI and connectedness, indicating a detectable difference between the odds ratios when comparing the NSSI and no NSSI groups.* *∗*p *< .05*

For all 4 models, the presence of NSSI was positively associated with screening positive for suicide risk, whereas an increase in connectedness (overall, school, family, and peer) was negatively associated with suicide risk (Table 3; Tables S2-S5, available online). The only significant interaction was between family connectedness and NSSI, where family connectedness was less protective for youth with NSSI compared with youth without NSSI (*z* = 1.96, *p* = .05). For every 1-point increase from mean family connectedness, the odds of screening positive for suicide risk decreased by 62% (odds ratio [OR] 0.38, 95% CI 0.31-0.46) for youth with NSSI and 70% (OR 0.30, 95% CI 0.27-0.34) for youth without NSSI (Figure 2).

**Table 3:** Main Effects of Each Logistic Regression Model

	*b*	SEM	*z*	*p*
Overall connectedness				
NSSI	2.71	0.10	25.96	∗∗∗
Overall connectedness	−1.32	0.07	−18.61	∗∗∗
NSSI × connectedness	.11	0.14	0.77	.44
Peer connectedness				
NSSI	3.02	0.09	32.46	∗∗∗
Peer connectedness	−.46	0.05	−9.69	∗∗∗
NSSI × connectedness	.04	0.09	0.45	.65
School connectedness				
NSSI	2.86	0.10	28.78	∗∗∗
School connectedness	−.74	0.05	−15.12	∗∗∗
NSSI × connectedness	.16	0.10	1.67	.10
Family connectedness				
NSSI	2.75	0.11	26.02	∗∗∗
Family connectedness	−1.20	0.06	−20.01	∗∗∗
NSSI × connectedness	.23	0.12	1.96	∗

Note: Only variables of interest are shown. All covariates are included in Tables S2, S3, S4, and S5, available online. NSSI = nonsuicidal self-injury.

∗*p* < .05; ∗∗∗*p* < .001.

For peer, school, and overall connectedness, there were no detectable differences in decreased odds of suicide risk between the NSSI and non-NSSI groups (Figure 2). For every 1-point increase from mean school connectedness, the odds of screening positive for suicide risk decreased by 44% (OR 0.56, 95% CI 0.48-0.66) for youth with NSSI and 52% (OR 0.48, 95% CI 0.43-0.53) for youth without NSSI. For every 1-point increase from mean peer connectedness, the odds of screening positive for suicide risk decreased by 34% (OR 0.66, 95% CI 0.57-0.76) for youth with NSSI and 37% (OR 0.63, 95% CI 0.57-0.69) for youth without NSSI. Finally, for every 1-point increase from mean overall connectedness, the odds of screening positive for suicide risk decreased by 70% (OR 0.30, 95% CI 0.23-0.38) for youth with NSSI and 73% (OR 0.27, 95% CI 0.23-0.31) for youth without NSSI. Additional plots for odds of screening positive for suicide risk depending on levels of connectedness are included in Figures S1 through S4, available online.

## Discussion

Higher levels of connectedness (overall, school, family, peer) were associated with decreased odds of screening positive for suicide risk in both youth who did and did not engage in NSSI. Contrary to our hypothesis, there were no significant interactions between overall, school, and peer connectedness with NSSI, with the exception of family connectedness, which had a weaker protective effect for the NSSI group. This indicates that connectedness also did not impact the correlation of NSSI with suicide risk, which may be due to the robust association between NSSI and suicide risk. These results are in line with previous analyses in the ED-STARS sample, where the protective effects of connectedness were lower for youth who had known suicide risk factors such as a lifetime attempt.^38^ A prior study also found that although protective factors such as resilience and subjective happiness were associated with decreased likelihood of suicide attempt, they did not moderate the association between NSSI frequency and suicide attempt.^39^

The protective effect of family connectedness was lower for youth who engaged in NSSI compared with youth who did not. As the 2-item measure assesses only the child’s perception of their family’s understanding and attention, a high connectedness score may not capture a family’s specific responses to NSSI and mental health. NSSI can negatively affect family dynamics, where caregivers can often feel stress, guilt, and confusion in response to a child who engages in self-injury.^40^ These responses may influence how family members support the child, potentially leading to controlling behaviors such as over-questioning, which can exacerbate NSSI severity. In these cases, increased family connectedness may be less protective due to the specific reaction of family members to NSSI behaviors.

All domains of connectedness offered protective effects against suicide risk regardless of the adolescent’s past-year NSSI history. These results emphasize the importance of family, school, and peer connectedness as potential intervention targets for suicide prevention in high-risk youth. Identifying which domain of connectedness could be strengthened in an adolescent could help clinicians better connect the patient to the right intervention and resource. Additionally, incorporating short family-centered interventions within the visit itself may reduce stress on patients and families.

Interventions in various clinical settings that improve family support and education have been shown to increase outpatient treatment adherence^41^ and decrease inpatient hospitalization rates,^42^ respectively, compared with treatment-as-usual conditions. In the ED, examples include the family intervention for suicide prevention, which includes a brief youth and family crisis therapy session and follow-up telephone contacts and the family-based crisis intervention, which includes a 60- to 90-minute session with the youth and family conducted by a social worker. Additionally, treatments such as attachment-based family therapy, which targets family processes such as cohesion, have also shown more efficacy in reducing suicidal ideation among youth in the ED compared with controls.^43^ In outpatient settings, treatments such as family centered brief intensive treatment can serve as an alternative for hospitalization and is more effective in reducing suicidality and hopelessness compared with outpatient therapy without the family component.^44^

These results also point to the value of community-level interventions in addition to individual-level interventions. Many school-based interventions focus primarily on identifying warning signs and coping skills, but do not enhance peer relationships or engage families, which can limit their effectiveness.^45^ Initiatives such as Sources of Strength, which uses a peer leadership model to target social connectedness and norms about suicide school-wide, have been shown to increase help-seeking behaviors especially in students with a history of suicidal ideation.^20^ These programs can be incorporated into a continuity-of-care model, where suicide prevention specialists help coordinate care for youth identified for suicide risk across multiple systems such as schools, community organizations, and hospitals, which facilitates the transition of care and decreases suicide attempts and ED visits.^46^

Findings from this study also revealed important information about underserved groups such as gender minority and multiracial adolescents. In this sample, gender minority adolescents had the highest rates of NSSI and suicide risk compared with cisgender male and cisgender female adolescents. This aligns with a growing body of research that shows gender minorities are at elevated risk for NSSI and STBs compared with cisgender youth.^47^^,^^48^ Compared with their monoracial peers, multiracial adolescents also had the highest rates of NSSI and suicide risk. Although multiracial people are the fastest growing population in the United States, there is limited research on this racial group. Studies that have examined mental health outcomes in this group show that they have higher levels of anxiety and depressive symptoms.^49^ These rates highlight the need for more research in these populations. Future research should disaggregate data to differentiate between sex and gender variables when possible and to allow individuals to report a multiracial identity or potentially differentiate the multiracial category into different subgroups to avoid overgeneralization.

Several limitations should be considered for this study. First, this was a sample of pediatric ED patients, so findings have limited generalizability to other youth populations and settings. Additionally, the design was cross-sectional, and determining temporality of NSSI and STBs was not possible. Thus, it is unclear if NSSI preceded suicidal ideation or behavior, limiting interpretability of NSSI as a predictor of suicide risk. Finally, school, family, and peer connectedness were assessed with only 2 items each, which may not comprehensively capture these constructs. The items also did not directly inquire if participants felt comfortable disclosing mental health concerns to others, so someone with high perceived connectedness could still feel unsupported regarding their NSSI behavior or suicidal ideation.

Future research should examine connectedness in greater detail such as the quantity and nature of different relationships and interventions that could be protective for at-risk youth. For example, few studies on youth suicide prevention interventions incorporate specific measures on family connectedness and changes in family dynamics, limiting our understanding of which aspects of the interventions (eg, increasing education vs improving relationships) may be most effective in reducing STBs.^50^ Additionally, future studies should evaluate the association between NSSI, suicide risk, and connectedness using a prospective design and in other medical settings such as outpatient clinics. There is also limited research on the interconnection between different domains of connectedness (eg, the effect of having low family connectedness, but high peer connectedness), and their relative contributions to decreased risk. Finally, as demographic variables such as race and gender were not examined in detail in the current study, additional research is needed to examine the effects of connectedness and NSSI in minoritized youth.

Connectedness showed a protective effect on screening positive for suicide risk that generally did not differ between youth who engaged in NSSI in the past year and youth who did not. System-level interventions that strengthen family, school, and peer networks that can be implemented before STBs emerge and reach a broad population of youth have the potential to reduce youth suicide.

## CRediT authorship contribution statement

**Tesia Shi:** Writing – review & editing, Writing – original draft, Visualization, Methodology, Formal analysis, Data curation, Conceptualization. **Ritika Merai:** Writing – review & editing, Methodology, Formal analysis, Conceptualization. **Nathan J. Lowry:** Writing – review & editing, Conceptualization. **Donna A. Ruch:** Writing – review & editing. **Jeffrey A. Bridge:** Writing – review & editing. **Maryland Pao:** Writing – review & editing, Supervision. **Lisa M. Horowitz:** Writing – review & editing, Supervision, Conceptualization.
