# Supplementary material for: The Relationships Between Nonsuicidal Self-Injury, Connectedness, and Suicide Risk in Youth Presenting to the Emergency Department
Source: JAACAP Open. 2025 Jan 14;3(3):439–47. doi: 10.1016/j.jaacop.2025.01.001 (PMC12414299; doi:10.1016/j.jaacop.2025.01.001)
Supplement: Supplemental Figures Tables [file mmc1.docx]

**Supplemental Materials**

**Table S1. Mean Connectedness Across Sample Demographics**

| **Demographics** | **Overall Connectedness**  **Mean (SD)** | **Peer Connectedness**  **Mean (SD)** | **School Connectedness**  **Mean (SD)** | **Family Connectedness**  **Mean (SD)** |
| --- | --- | --- | --- | --- |
| **Sample** | 3.94 (0.74) | 4.02 (0.96) | 3.70 (0.99) | 4.10 (0.87) |
| **Gender**  Male  Female  Gender Minority | 4.07 (0.66)  3.86 (0.77)  3.22 (0.87) | 4.08 (0.88)  3.98 (1.01)  3.66 (1.15) | 3.87 (0.89)  3.60 (1.02)  2.76 (1.27) | 4.26 (0.78)  4.01 (0.90)  3.23 (0.94) |
| **Race/Ethnicity** |  |  |  |  |
| Hispanic or Latino | 3.92 (0.73) | 3.92 (0.98) | 3.69 (0.95) | 4.15 (0.87) |
| NH AANHPI | 4.05 (0.59) | 4.12 (0.82) | 3.79 (0.88) | 4.23 (0.79) |
| NH AIAN | 3.88 (0.78) | 3.76 (0.99) | 3.61 (1.08) | 4.26 (0.82) |
| NH Black | 3.86 (0.74) | 3.84 (1.01) | 3.66 (0.97) | 4.08 (0.91) |
| NH Multiracial | 3.84 (0.77) | 3.96 (0.97) | 3.62 (0.99) | 3.93 (0.93) |
| NH White | 4.00 (0.75) | 4.17 (0.91) | 3.73 (1.03) | 4.10 (0.85) |
| **Chief Complaint**  Medical  Psychiatric  Unknown | 4.01 (0.69)  3.79 (0.82)  5 | 4.06 (0.92)  3.94 (1.04)  5 | 3.77 (0.94)  3.55 (1.08)  5 | 4.20 (0.80)  3.90 (0.96)  5 |

Note: AANHPI = Asian American Native Hawaiian Pacific Islander, AIAN = American Indian/Alaska Native, NH = non-Hispanic

**Table S2. Logistic Regression Model Values for Overall Connectedness**

|  | **b** | **Standard Error** | **Z value** | **p - value** |
| --- | --- | --- | --- | --- |
| **Main Effects**  NSSI  Overall Connectedness  NSSI x Connectedness | 2.71  -1.31  0.11 | 0.10  0.07  0.14 | 25.96  -18.61  0.77 | ***  ***  0.44 |
| **Gender**  Male  Female  Gender Minority | Reference  0.64  1.41 | Reference  0.09  0.29 | Reference  6.92  4.80 | Reference  ***  *** |
| **Race/Ethnicity** |  |  |  |  |
| NH White | Reference | Reference | Reference | Reference |
| Hispanic or Latino | -0.29 | 0.11 | -2.69 | ** |
| NH AANHPI | 0.24 | 0.33 | 0.72 | 0.47 |
| NH AIAN | -0.60 | 0.55 | -1.08 | 0.28 |
| NH Black | -0.18 | 0.11 | -1.67 | 0.1 |
| NH Multiracial | -0.15 | 0.21 | -0.73 | 0.47 |
| **Age** | 0.05 | 0.03 | 1.90 | 0.06 |

Note: ∗p < .05; ∗∗p < .01; ∗∗∗p < .001, AANHPI = Asian American Native Hawaiian Pacific Islander, AIAN = American Indian/Alaska Native, NH = non-Hispanic

**Table S3. Logistic Regression Model Values for Peer Connectedness**

|  | **b** | **Standard Error** | **Z value** | **p - value** |
| --- | --- | --- | --- | --- |
| **Main Effects**  NSSI  Peer Connectedness  NSSI x Connectedness | 3.02  -0.46  0.04 | 0.09  0.05  0.09 | 32.46  -9.69  0.45 | ***  ***  0.65 |
| **Gender**  Male  Female  Gender Minority | Reference  0.76  1.83 | Reference  0.09  0.27 | Reference  8.57  6.69 | Reference  ***  *** |
| **Race/Ethnicity**  NH White | Reference | Reference | Reference | Reference |
| Hispanic or Latino | -0.29 | 0.1 | -2.73 | ** |
| NH AANHPI | 0.18 | 0.32 | 0.56 | 0.58 |
| NH AIAN | -0.54 | 0.51 | -1.05 | 0.3 |
| NH Black | -0.12 | 0.11 | -1.1 | 0.27 |
| NH Multiracial | -0.08 | 0.2 | -0.42 | 0.67 |
| **Age** | 0.10 | 0.03 | 3.88 | *** |

Note: ∗p < .05; ∗∗p < .01; ∗∗∗p < .001, AANHPI = Asian American Native Hawaiian Pacific Islander, AIAN = American Indian/Alaska Native, NH = non-Hispanic

**Table S4. Logistic Regression Model Values for School Connectedness**

|  | **b** | **Standard Error** | **Z value** | **p - value** |
| --- | --- | --- | --- | --- |
| **Main Effects**  NSSI  School Connectedness  NSSI x Connectedness | 2.86  -0.74  0.16 | 0.10  0.05  0.10 | 28.78  -15.12  1.67 | ***  ***  0.10 |
| **Gender**  Male  Female  Gender Minority | Reference  0.66  1.58 | Reference  0.09  0.29 | Reference  7.40  5.46 | Reference  ***  *** |
| **Race/Ethnicity**  NH White | Reference | Reference | Reference | Reference |
| Hispanic or Latino | -0.19 | 0.11 | -1.78 | 0.08 |
| NH AANHPI | 0.22 | 0.33 | 0.67 | 0.51 |
| NH AIAN | -0.49 | 0.56 | -0.89 | 0.38 |
| NH Black | -0.02 | 0.11 | -0.14 | 0.89 |
| NH Multiracial | -0.02 | 0.2 | -0.11 | 0.91 |
| **Age** | 0.07 | 0.03 | 2.61 | ** |

Note: ∗p < .05; ∗∗p < .01; ∗∗∗p < .001, AANHPI = Asian American Native Hawaiian Pacific Islander, AIAN = American Indian/Alaska Native, NH = non-Hispanic

**Table S5. Logistic Regression Model Values for Family Connectedness**

|  | **b** | **Standard Error** | **Z value** | **p - value** |
| --- | --- | --- | --- | --- |
| **Main Effects**  NSSI  Family Connectedness  NSSI x Connectedness | 2.75  -1.20  0.23 | 0.11  0.06  0.12 | 26.02  -20.01  1.96 | ***  ***  * |
| **Gender**  Male  Female  Gender Minority | Reference  0.63  1.31 | Reference  0.09  0.29 | Reference  6.83  4.50 | Reference  ***  *** |
| **Race/Ethnicity**  NH White | Reference | Reference | Reference | Reference |
| Hispanic or Latino | -0.15 | 0.11 | -1.39 | 0.16 |
| NH AANHPI | 0.31 | 0.34 | 0.92 | 0.36 |
| NH AIAN | -0.18 | 0.55 | -0.33 | 0.74 |
| NH Black | -0.05 | 0.11 | -0.4 | 0.69 |
| NH Multiracial | -0.12 | 0.21 | -0.60 | 0.55 |
| **Age** | 0.06 | 0.03 | 2.04 | * |

Note: ∗p < .05; ∗∗p < .01; ∗∗∗p < .001, AANHPI = Asian American Native Hawaiian Pacific Islander, AIAN = American Indian/Alaska Native, NH = non-Hispanic


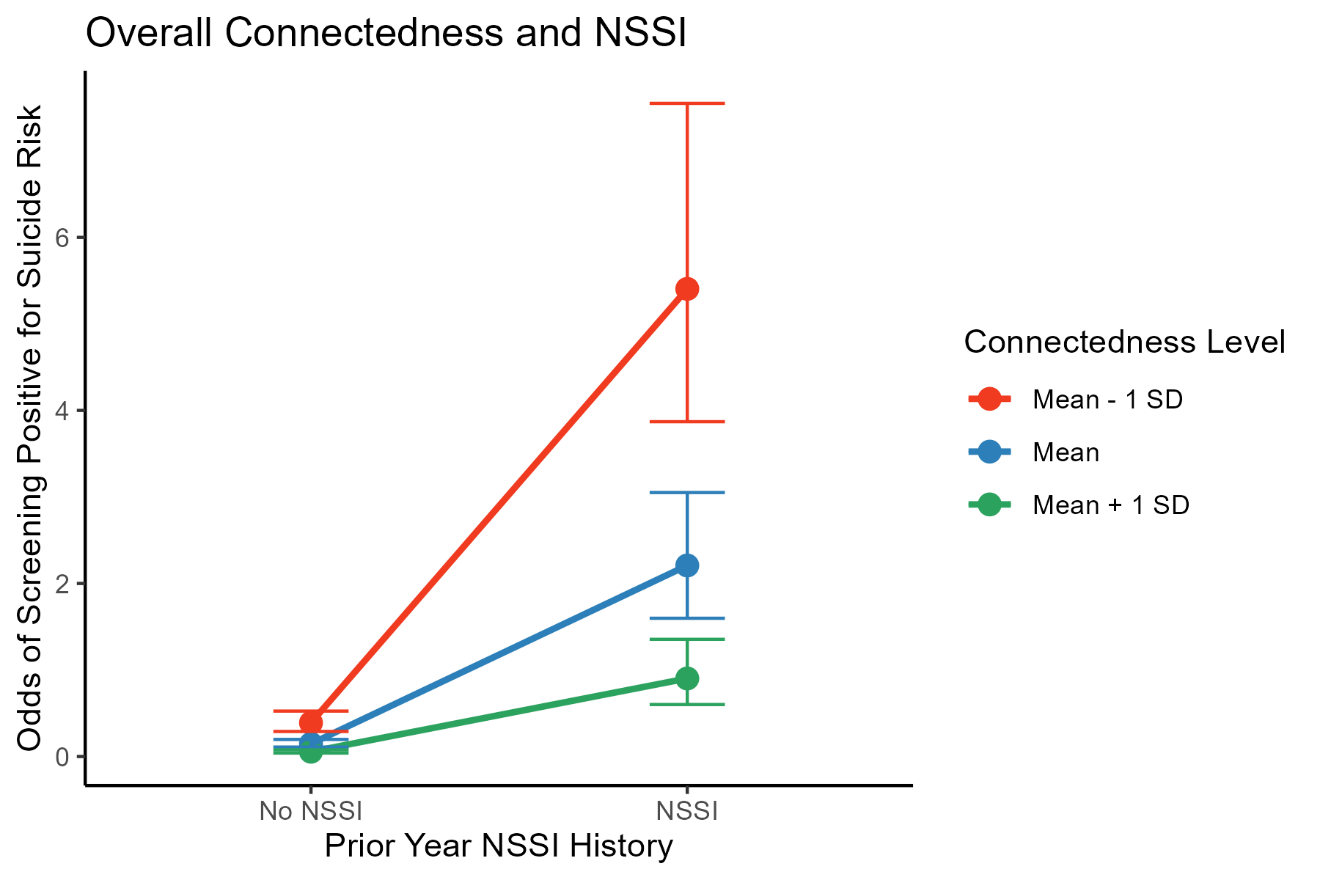


**Figure S1. Odds of screening positive for suicide risk by levels of overall connectedness and NSSI**

Note: SD = standard deviation

**
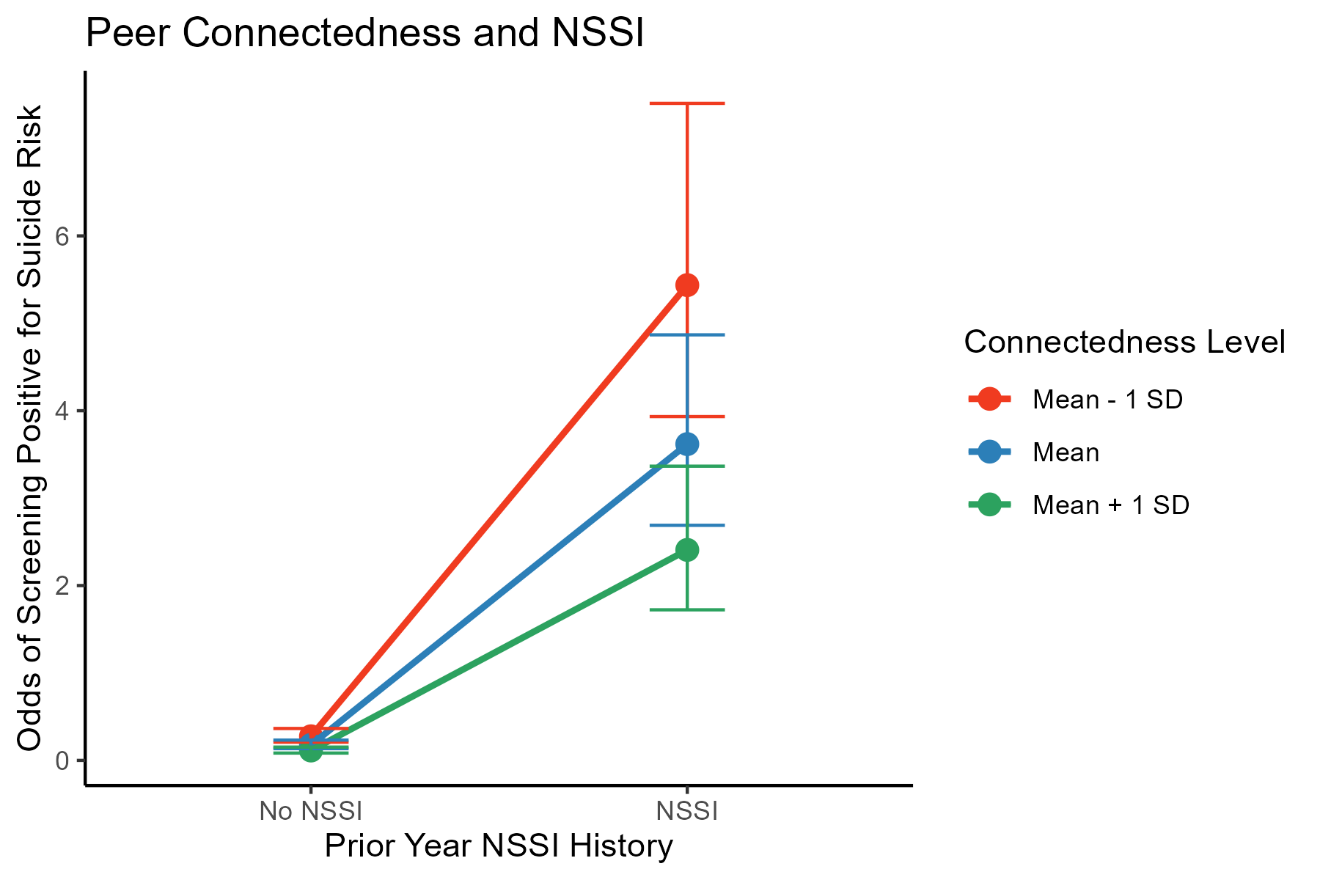
**

**Figure S2. Odds of screening positive for suicide risk by levels of peer connectedness and NSSI**

Note: SD = standard deviation

**
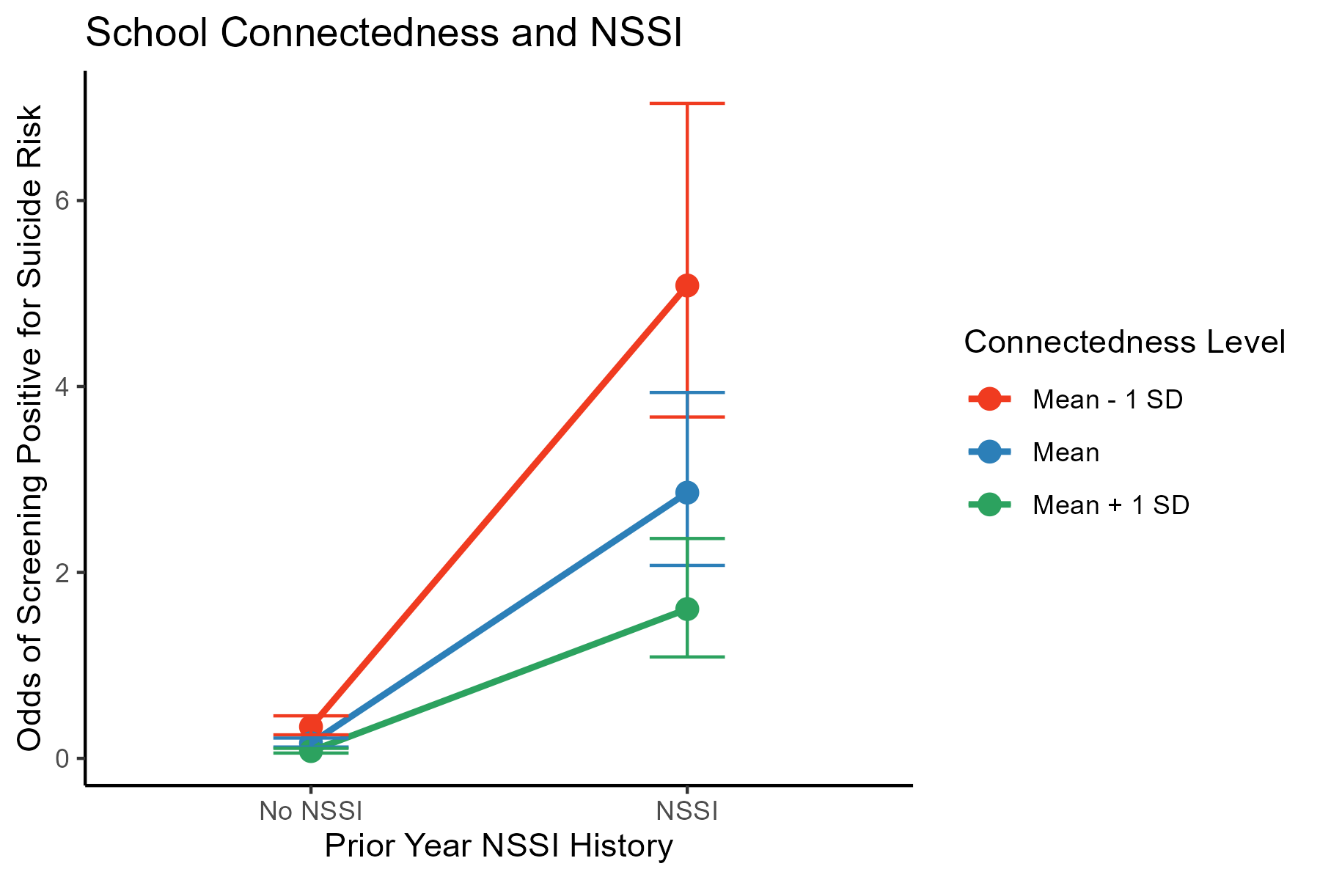
**

**Figure S3. Odds of screening positive for suicide risk by levels of school connectedness and NSSI**

Note: SD = standard deviation

**
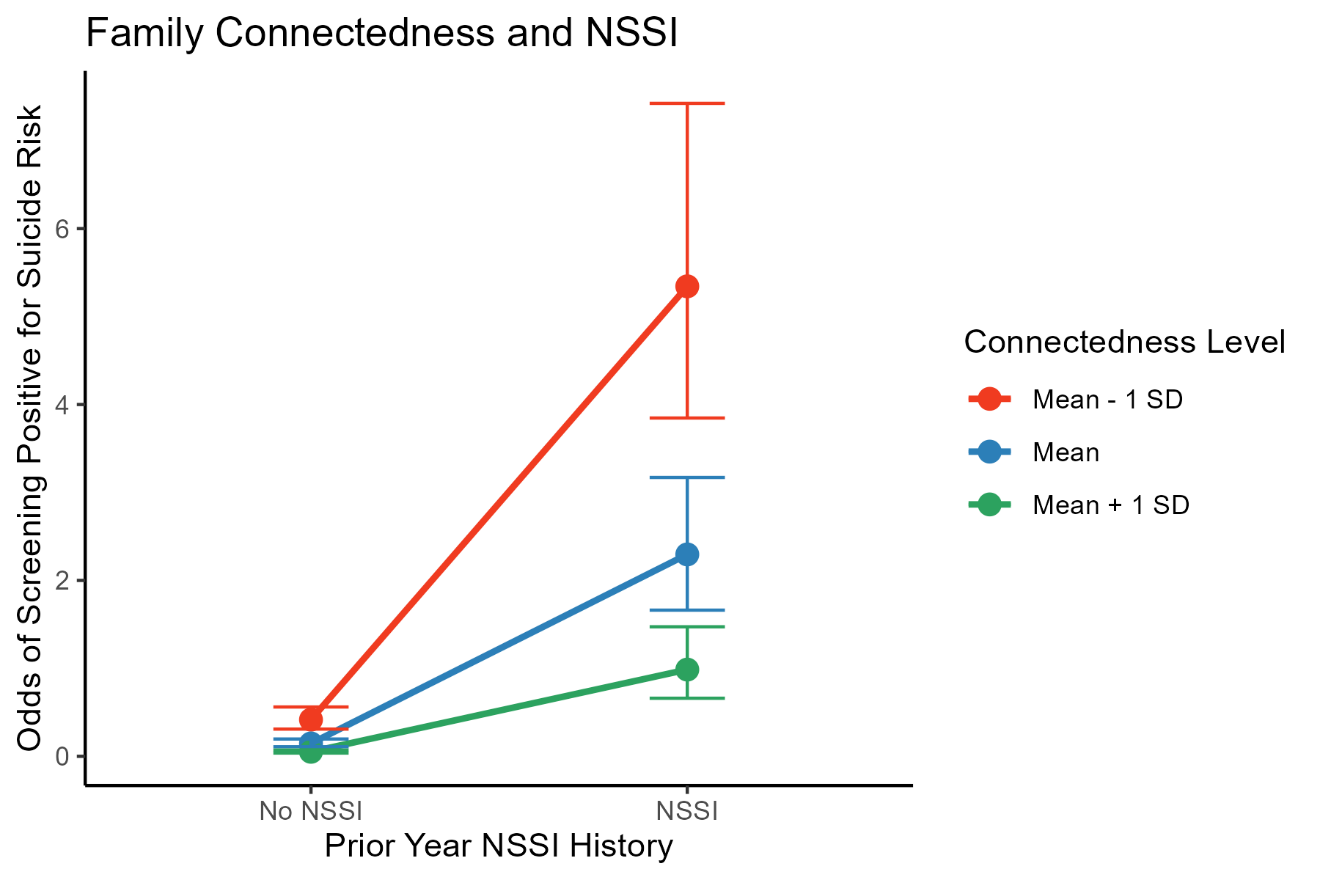
**

**Figure S4. Odds of screening positive for suicide risk by levels of family connectedness and NSSI**

Note: SD = standard deviation
